# Operation of Wearable Thermoelectric Generators Using Dual Sources of Heat and Light

**DOI:** 10.1002/advs.202104915

**Published:** 2022-02-24

**Authors:** Myeong Hoon Jeong, Kwang‐Chon Kim, Jin‐Sang Kim, Kyoung Jin Choi

**Affiliations:** ^1^ Department of Materials Science and Engineering Ulsan National Institute of Science and Technology Ulsan 44919 Republic of Korea; ^2^ Center for Electronic Materials Korea Institute of Science and Technology Seoul 02792 Republic of Korea; ^3^ KIST Jeonbuk Institute of Advanced Composite Materials Wanju‐gun 55324 Republic of Korea

**Keywords:** BiTe, high temperature difference, printing, solar absorber, thermoelectric, wearable

## Abstract

A wearable thermoelectric generator (WTEG) that utilizes human body heat can be a promising candidate for the wearable power generators. The temperature difference (Δ*T*) between the body and the environment is a stable source driving the WTEG, but this driving force is limited by the ambient temperature itself at the same time. Here, a novel WTEG that can be operated using the dual source of body heat and light with exceptionally high driving force is fabricated. The printable solar absorbing layer attached to the bottom of the WTEG absorbs ≈95% of the light from ultraviolet to far infrared and converts it into heat. To optimize the power density of WTEGs, the fill factor of the thermoelectric (TE) leg/electrode is considered through finite‐difference time‐domain (FDTD) simulation. When operated by the dual sources, the WTEG exhibits a power density of 15.33 µW cm^−2^, which is the highest under “actual operating conditions” among all kinds of WTEGs. In addition, unlike conventional WTEGs, the WTEG retains 83.1% of its output power at an ambient temperature of 35 °C compared to its output power at room temperature. This study will accelerate the commercialization of WTEGs by introducing a novel method to overcome their limitations.

## Graphical abstract

Vertical‐type wearable thermoelectric generator (WTEG) driven by light and body heat simultaneously is proposed first by applying a solar absorbing layer. The WTEG overcomes the issue of low temperature difference (Δ*T*) and output power from conventional WTEGs that harvest energy from body heat. Among the reported WTEGs attached to human skin, the highest output power density is realized.

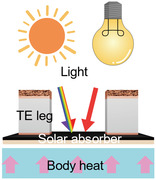

## 1 Introduction

Recently, wearable electronics have made remarkable progress, and products such as smartwatches and wireless earphones have already been commercialized. Since most wearable electronics are not self‐powered systems, they need to be periodically charged, and the weight or size of the products comes mainly from the battery. Due to these problems, the importance of self‐powered wearable systems is emerging. Wearable thermoelectric generators (WTEGs) can be good energy harvesting systems for wearable electronics since they are driven by the temperature difference (Δ*T*) between the human body and the ambient temperature.^[^ ^1^ ^]^

WTEG can be divided into lateral and vertical structures depending on the shape of the thermoelectric (TE) leg. Lateral type WTEGs with the TE legs arranged laterally along the substrate are fabricated by printing,^[^ ^2^, ^3^, ^4^, ^5^ ^]^ coating,^[^ ^6^, ^7^, ^8^, ^9^, ^10^ ^]^ or depositing^[^ ^11^, ^12^ ^]^ flexible organic TE materials such as Poly(3,4‐ethylenedioxythiophene) : poly(styrenesulfonate) (PEDOT:PSS),^[^ ^6^, ^13^, ^14^, ^15^, ^16^ ^]^ carbon nanotube (CNT) composites,^[^ ^17^, ^18^, ^19^, ^20^, ^21^ ^]^ fullerene derivatives,^[^ ^22^, ^23^, ^24^ ^]^ or TE inks made of rigid inorganic TE nanoparticles on flexible substrates.^[^ ^11^, ^12^ ^]^ Lateral type WTEGs with long and thin TE legs have high Δ*T*. However, the low dimensionality of TE legs also causes high electrical resistance, resulting in low power densities on the order of nW cm^−2^. For example, Cao et al. fabricated flexible TEG by screen printing eight pairs of BiTe/SbTe TE legs and reported an output power of 444 nW at a Δ*T* of 20 °C and internal electrical resistance of 1.7 kΩ.^[^ ^4^ ^]^

On the other hand, vertical type WTEGs have a structure in which rigid inorganic TE pellets are vertically aligned between two substrates to allow vertical heat flow through the legs. They also have a soft polymer infiltrated between TE legs to give flexibility to the devices.^[^ ^25^ ^]^ Inorganic bulk TE pellets such as Bi_2_Te_3_, Sb_2_Te_3_, and GeTe have higher ZT performance and low electrical resistance,^[^ ^26^, ^27^, ^28^, ^29^, ^30^ ^]^ so vertical type WTEGs tend to have tens to hundreds of times higher output power density than lateral type WTEG.^[^ ^31^, ^32^, ^33^, ^34^, ^35^, ^36^, ^37^, ^38^ ^]^ However, the low Δ*T* still acts as a major obstacle in vertical devices, which is partly due to the dimension of the TE legs or inefficient WTEG structure.^[^ ^27^, ^31^, ^39^, ^40^ ^]^ For example, a long TE leg is advantageous in achieving high Δ*T*, but on the other hand, it increases the internal resistance, which offsets the advantage of high Δ*T*.^[^ ^41^ ^]^ Additionally, the flexibility of the device can be compromised by the long TE legs, so the appropriate leg length is 1–2 mm in vertical devices taking all these factors into account.^[^ ^42^ ^]^ In addition, limited body heat also causes the low Δ*T* of body heat‐driven WTEGs.^[^ ^43^ ^]^ Human skin is thermally insulating and insensitive to the environment. Although the amount of heat generated varies slightly depending on the body part, the human body generates a constant temperature of 36 °C. This characteristic of the human body can be an advantage of stably providing a heat source but a disadvantage of having limited maximum power. Since all types of WTEGs are driven by the Δ*T* between the body temperature and ambient temperature, a sustainable Δ*T* of up to 10 °C can be obtained at room temperature. However, only a small fraction of the Δ*T* contributes to the output power of the WTEG due to the low thermal resistance, and even the Δ*T* disappears as the ambient temperature approaches the body temperature.^[^ ^44^ ^]^

In this study, we propose, for the first time a vertical WTEG operated by the dual‐source of heat and light to provide a solution to the low‐Δ*T* issue of the WTEG. The polysiloxane‐based light absorbing layer printed on the bottom side of the WTEG harvests light in a wide range of wavelengths from ultraviolet to far infrared with an absorbance of >95%. The WTEG can be operated in three modes, namely, body‐heat, sunlight, and dual‐source‐operation modes, depending on the surrounding environment. The WTEG generates Δ*T* values of 5.5, 9.2, and 10.9 °C in body‐heat, sunlight, and dual‐source‐operation modes, respectively. As a result, power densities of 2.19, 8.28, and 15.33 µW cm^−2^ are obtained depending on the modes, respectively. In other words, the power density obtained in the dual‐source‐operation mode is sevenfold higher than that in the body‐heat‐operation mode and corresponds to a record‐high value among WTEGs based on all types of TE materials and device structures. Furthermore, unlike most body‐heat‐driven WTEGs, our device produces a high‐power density even at an ambient temperature of 37 °C.

## 2 Results and Discussions

### 2.1 WTEG Fabrication

**Figure 1:**
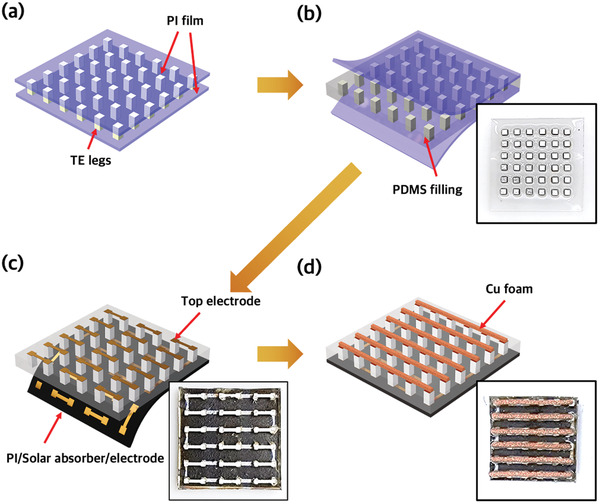
Schematic illustration and photographs of the fabrication process for sunlight‐assisted WTEGs. a) p‐n TE leg pairs are alternately aligned between PI films. b) PDMS is inserted into the gap between the two PI films. After curing PDMS, the PI films are peeled off. c) Solar absorbers are printed on the PI film, dumbbell‐shaped electrodes are deposited on the top of PDMS and the PI/solar absorber film and successively attached to the bottom of PDMS. The p‐n TE leg pairs are connected in series. d) Cu foam is attached for cooling. It has the same width as the thinnest part of the dumbbell‐shaped electrode to prevent the copper foam from blocking the solar absorber.

**Figure** **1** illustrates the fabrication process of the WTEGs. The structure of the device is similar to vertical WTEGs, except that a solar absorbing layer is integrated into the bottom of the device. First, p‐n TE legs with a height of 1.5 mm and various cross‐sectional areas were alternately aligned at calculated intervals between two polyimide (PI) films (Figure 1a). A polydimethylsiloxane (PDMS) elastomer was inserted between two PI films and cured, forming a flexible TE leg polymer array structure (Figure 1b). The insertion of a soft medium, e.g., PDMS, provides an acceptable mechanical tolerance to rigid TEGs. After the PI films were peeled off, 4‐µm‐thick Ag top electrodes were deposited through a shadow mask on the top of PDMS using e‐beam evaporation. Ni (50 nm) was pre‐deposited on the PDMS to improve the adhesion between the Ag and PDMS. The solar absorbing layer was prepared by painting silicone‐based solar absorbing paint with a solar absorption of 95% on the PI film.^[^ ^45^ ^]^ After the Ag bottom electrodes were deposited on the solar absorbing film/PI substrate using e‐beam evaporation, the composite structure of the Ag electrode/solar absorber/PI substrate was attached to the bottom of the PDMS while aligning the electrode and TE leg and electrically connecting them together with highly conductive silver paste (Figure 1c). The electrode was designed in a dumbbell shape by reducing the width of the electrode between two adjacent TE legs. Compared to rectangular shapes, dumbbell‐shaped electrodes can decrease the fill factor, allowing the solar absorber to have increased exposure to sunlight and generate more heat without significant electrical resistance loss. As shown in Figure S1b (Supporting Information), in the case of rectangular shapes, fill factors were 19–42% depending on the leg size, but they decreased to 11–28% when dumbbell‐shaped electrodes were introduced. Accordingly, the hot side temperature increased in all leg sizes, and the Δ*T* increased by 1.1–2.6 °C (Figure S1c–d, Supporting Information). Finally, a copper foam heat sink with high thermal conductivity and a large surface area was integrated to accelerate heat dissipation on the cold side. Since vertical‐type WTEGs have difficulty dissipating heat that is quickly conducted through the TE leg, the use of a heat sink can be effective in improving the Δ*T* and power. The heat sink is designed to maximize its surface area to maximize its convective heat transfer to the surroundings.^[^ ^46^ ^]^ However, it is challenging to introduce a heat sink wider than the device or to attach fins to the WTEG. Copper foam is an optimal material as a heat sink for WTEGs due to its large surface area and flexibility.^[^ ^40^ ^]^ They have the same width as the thinnest part of the dumbbell‐shaped electrode to prevent the copper foam from blocking the solar absorber.

### 2.2 Systematic Optimization of the WTEG Design

Since the output voltage and internal electrical resistance are changed depending on the device structure, an optimized WTEG structure is required. In general, the amount of TEG power is given by the following equation:^[^ ^43^, ^47^ ^]^

(1) $$P_{max}={\left(N\times \left(\alpha_{n}+\alpha_{p}\right)\Delta T\right)}^{2}\;/R_{i}{\left(1+R_{L}/R_{i}\right)}^{2}$$

where *N* is the number of p‐n TE leg pairs, *α_n_* and *α_p_* are the Seebeck coefficients of the n‐type and p‐type legs, respectively, *R_i_* is the internal electrical resistance, and *R_L_* is the loaded electrical resistance. As given in Equation (1), since the numerator and denominator represent the output voltage and electrical resistance factor, respectively, the output power is proportional to the square of the voltage and is inversely proportional to the electrical resistance. The voltage and electrical resistance of the WTEG are mainly determined by the dimension of the TE leg and the number of TE legs. For example, Δ*T* applied to the legs is affected by the thermal resistance of the TE leg, which changes with the fill factor (the number of TE legs × the cross‐sectional area of a single TE leg + area of the electrode) of the TE leg/electrode and further results in a change in voltage.^[^ ^47^ ^]^ On the other hand, increasing the number of TE legs can improve the output voltage in series connection mode; However, the internal electrical resistance of the TEG increases as well, which results in poor output power. Due to these complex relationships, it is challenging to finalize the WTEG structure with the maximum output power only experimentally. Therefore, it is necessary to systematically calculate the required number and cross‐sectional area of the legs to obtain the maximum power. The Δ*T* and output power according to the WTEG structure were analyzed, and an optimal structure was predicted using COMSOL simulation.

**Figure 2:**
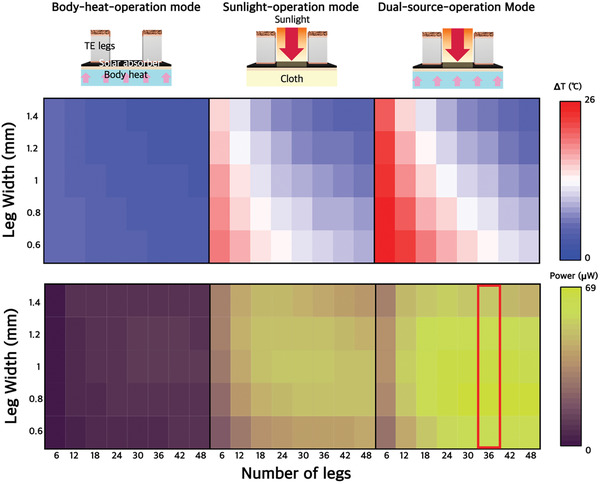
Simulated Δ*T* and power mapping of a WTEG in body‐heat‐operation mode, sunlight‐operation mode, and dual‐source‐operation mode depending on the leg area and number of legs. Body‐heat‐operation mode: Only body heat is harvested. Sunlight‐operation mode: The solar absorbing layer harvests additional heat from solar radiation, increasing Δ*T*. However, a significant amount of heat dissipates from the bottom. Dual‐source‐operation mode: The WTEG attached to the skin generates a considerable amount of heat when exposed to sunlight and can maintain a high temperature due to the low thermal conductivity of skin.

**Figure** **2** shows a mapping of the calculated Δ*T* depending on the fill factor of the leg and electrode while keeping the leg height constant at 1.5 mm. To observe the effect of the solar absorber, we compared Δ*T* and output power changes according to three different conditions. The body‐heat‐operation mode represents the condition driven by body temperature, similar to the conventional WTEG. In body‐heat‐operation mode, the lowest Δ*T* (≈2.2 °C) is obtained when the fill factor is the highest (the width of the leg is 1.4 mm, and the number of legs is 48), and the highest Δ*T* (≈6.3 °C) is obtained when the fill factor is the lowest (width of the leg is 0.6 mm, and the number of legs is 6). These results have the same trend as that of conventional WTEGs driven by body heat. Thermodynamically, the human body is a low‐temperature heat reservoir (*T*_body_ ≈ 36 °C) that emits thermal energy at a rate of ≈25 mW cm^−2^ into the ambient atmosphere,^[^ ^27^ ^]^ making practical application difficult because the conversion efficiency of WTEG is lower than 1%. The sunlight operation mode represents the condition in which the WTEG is attached to clothes and driven by sunlight only. Since the average sunlight intensity is 100 mW cm^−2^, the WTEG can absorb much more energy than when the WTEG is in the body‐heat‐operation mode. In sunlight‐operation mode, the relationship between the total area of the WTEG and Δ*T* becomes more complex. Under 1 sun illumination, the total solar power provided by the solar absorber is as follows:

(2) $$Q_{SA}=\;100mW/cm^{2}\times A\times \;\left(1-f\right)\;\times AR$$

where 100 mW cm^−2^ is the sunlight intensity under 1 sun illumination, *A* is the area of the solar absorber, *f* is the total fill factor of the TE leg and electrode, (1 – *f*) is the ratio of the solar absorber exposed to sunlight, and *AR* is the average absorption ratio of the solar absorber. The total fill factors according to the number and cross‐sectional area of the legs are summarized in Figure S2 (Supporting Information). Since printable solar absorbers can absorb an average of 95% of the available sunlight, if there are no TE legs and electrodes, the solar absorber theoretically obtains 380 mW of sunlight for a WTEG with an area (*A*) of 4 cm^2^. This solar energy is converted into heat, increasing Δ*T*. As the total fill factor increases, the amount of absorbed solar energy decreases. In short, the fill factor affects not only the thermal resistance but also solar energy absorption, thereby affecting Δ*T*. In sunlight‐operation mode, as shown in Figure 2, when six TE legs with a leg width of 0.6 mm are integrated into the device, the highest Δ*T* of ≈19.5 °C is observed, which is hard to obtain in conventional WTEGs, and when the fill factor is the highest (48 TE legs, 1.4 mm leg width), a Δ*T* of ≈4.9 °C can be obtained. However, in sunlight operation mode, the heat generated at the solar absorber is transferred not only to the TE leg but also to the fabric at a low temperature, which causes significant heat loss. In dual‐source‐operation mode, when WTEG is attached to the skin with sunlight irradiation, the body heat assists in heat generation due to intrinsic high temperature, allowing the WTEG to achieve a higher Δ*T*. In addition, due to the low thermal conductivity of human skin (*κ* ≈ 0.3 W m^−1^ K^−1^), the heat generated from the solar absorber can be kept from escaping to the skin. As a result, compared with the sunlight‐operation mode, the Δ*T* increased by 0.5–4 °C. Figure S4 (Supporting Information) shows the open‐circuit voltage (V_OC_) according to the number of legs. Due to the high Δ*T*, the highest voltage was obtained in the dual‐source‐operation mode. Additionally, since the TE legs were connected in series, in all modes, the *V*_OC_ increased as the number of TE legs increased. However, a high *V*_OC_ does not always lead to high power because increasing the number of legs will increase the internal electrical resistance, as mentioned above. Figure S6 (Supporting Information) shows the internal electrical resistance of the WTEG depending on the cross‐sectional area and number of legs. The internal electrical resistance is inversely proportional to the cross‐sectional area of the leg (Figure S6a, Supporting Information). Additionally, the internal electrical resistance increases linearly as the number of legs increases since not only the electrical resistance of the TE leg itself but also the electrical resistance of the junction and electrode increase in proportion to the number of legs (Figure S6b, Supporting Information). Considering all the results, the optimal calculated device power in dual‐source‐operation mode is 68.25 µW, and the power density is 17.06 µW cm^−2^ when 36 TE legs with a width of 0.8 mm are integrated.

### 2.3 Measuring WTEG Performance

**Figure 3:**
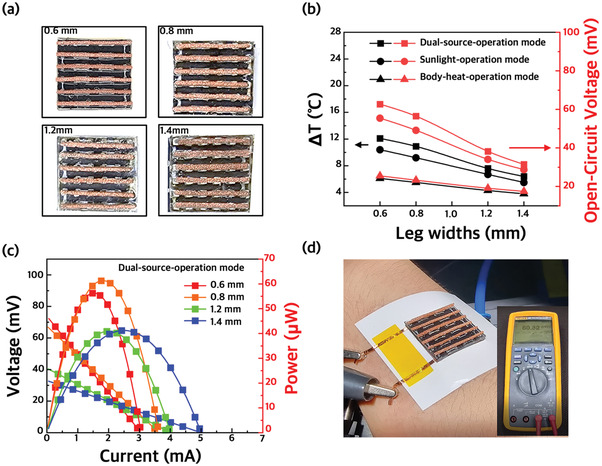
a) Photograph showing 18 n‐p TE leg pairs embedded into TEGs. TE legs with widths of 0.6, 0.8, 1.2, and 1.4 mm are used. b) Measured Δ*T* and open‐circuit voltage of the TEGs as a function of the fill factor. c) Measured TEG performance including voltage and power as a function of current. d) Photograph of measuring the voltage by placing the TEGs on human skin.

Through simulation results, we narrowed down candidates for TE leg conditions for optimal output power. We measured the power by fabricating a WTEG (leg width = 0.8 mm, number of legs = 36) to obtain the highest power based on the dual‐source‐operation mode. Additionally, WTEGs with widths of 0.6, 1.2, and 1.4 mm were fabricated to prove the Δ*T* or power change according to the leg width. **Figure** **3**a shows photos of WTEGs with various cross‐sectional areas of the legs. Figure 3b shows Δ*T* and *V*_OC_ of the WTEGs with various leg widths or three different modes. For stable measurement conditions, instead of attaching to the skin, the device measurement was performed on the thermally insulating film with a thermal conductivity of 0.3 W m^−1^ K^−1[^ ^44^ ^]^ and a temperature of 35 °C at an ambient temperature of 25 °C. In body‐heat‐operation mode, a Δ*T* of 4–6 °C was obtained depending on the leg width; a small *V*_OC_ of 17.5–25.5 mV was generated. As the simulation predicted, Δ*T* and *V*_OC_ slightly decreased as the leg width increased due to decreased thermal resistance. In sunlight‐operation mode, the WTEG was measured after placing on the fabric under 1 sun irradiation. The Δ*T* and *V*_OC_ were much higher because the solar absorber harvested sunlight and generated more heat. When the leg width was 0.6 mm, a Δ*T* of 10.4 °C was obtained, which is a remarkably high result. This result proves that WTEGs can be operated even when attached to clothes instead of in situations where the WTEG is attached to the skin, which other reported WTEGs could not. However, in sunlight‐operation mode, the fiber hardly transfers body temperature to the WTEG, so the WTEG is driven almost by solar energy only. On the other hand, WTEGs can harvest more energy in the dual‐source‐operation mode because the WTEG is attached to the body directly during sunlight irradiation. The WTEG obtained the highest Δ*T* and *V*_OC_ of 12.1 °C and 62.73 mV, respectively, when the leg width was 0.6 mm. The Δ*T* and *V*_OC_ were the highest when the leg width was 0.6 mm regardless of the mode, which matches the simulation results. As the width of the TE legs increased from 0.6 to 1.4 mm, the fill factor increased from 11% to 28%, screening the solar absorber layer more. However, as mentioned above, when the cross‐sectional area of the legs decreases, the temperature increases; however, the internal electrical resistance increases as well, resulting in low power. Figure 3c shows a comparison of the current–voltage output characteristics of the WTEGs with various leg widths in dual‐source‐operation mode. The output power was measured by changing the load resistances, and the maximum output power of the WTEG was obtained when the load resistance matched the internal resistance. As a result, we obtained the highest output power of 61.31 µW and power density of 15.33 µW cm^−2^ from the WTEG with a TE leg width of 0.8 mm in dual‐source‐operation mode. This result is remarkably higher compared to the output power (8.76 µW) of the WTEG in the body‐heat‐operation mode and that (33.12 µW) of the WTEG in the sunlight‐operation mode (Figure S5, Supporting Information). It is because that the Δ*T* of the WTEG in the body‐heat‐operation mode and the sunlight‐operation mode is 5.5 and 9.2 °C, respectively, which is lower than Δ*T* (10.9 °C) of the WTEG in dual‐source‐operation mode. As the TE leg width of the WTEG is increased to 0.6, 0.8, 1.2, and 1.4 mm, the *V*_OC_ gradually decreases to 59.09, 53.87, 31.44, and 27.66 mV. In contrast, as the width of the TE leg is increased, the *I*_SC_ increases to 3.02, 3.56, 3.96, and 4.95 mA, respectively, resulting from the decrease in internal electrical resistance. This result indicates that the simulated and experimental results are accurate and reliable. When the performance of the WTEG (0.8 mm TE leg width) was measured by attaching it to an arm, an output voltage of 55–62 mV was measured, which proves that the WTEG can be operated under practical conditions (Figure 3d).

**Figure 4:**
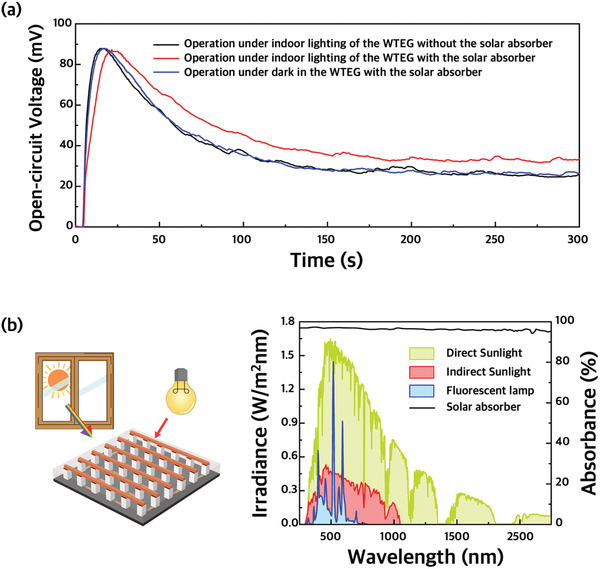
a) Measurement of *V*_OC_ of WTEG over time. When measured indoors during the day, the solar absorber harvests a small amount of scattered sunlight and generates a higher voltage. b) Schematic illustration of the heating processes of WTEG with a solar absorber under indirect sunlight and artificial light (left). Measured absorbance of the solar absorber and spectral irradiance of direct sunlight (yellow), indirect sunlight (red), and fluorescent lamp (blue) (right).

Figure S7 (Supporting Information) shows the *V*_OC_ of the WTEG as a function of time when the WTGE is attached to the arm. As soon as the WTEG is attached to the skin, the voltage rapidly increases for a few seconds and then decreases gradually to ≈30 mV (body‐heat‐operation mode). When exposed to sunlight, the WTEG shifts to dual‐source‐operation mode, the *V*_OC_ quickly increases to 130–140 mV in a few seconds, gradually decreases, and then eventually converges to ≈60 mV. After removing the sunlight, the voltage decreased rapidly and converged to ≈30 mV, the same voltage before sunlight irradiation. Interestingly, we found that the voltage of the WTEG with the solar absorber increases by 5–10 mV even when sunlight is not directly illuminated on the WTEG (**Figure** **4**a). As the *V*_OC_ of the WTEG without the solar absorber also decreases, it seems that the solar absorber harvests sorts of indirect sunlight from the outside or artificial lights such as fluorescent lamps; thus, the Δ*T* slightly increases. It is known that there is some scattered sunlight (≈227 W m^−2^) or artificial light (≈73 W m^−2^, fluorescent lamp) in an indoor environment (Figure 4b). Since the solar absorber has a high absorbance of over 95% in all wavelength ranges, it can collect even small amounts of light energy. As a result, it is confirmed that the WTEG with the solar absorber layer always shows higher performance even in the absence of direct sunlight. We expect this result to be extended to indoor applications in the future.

### 2.4 Ambient Temperature‐Insensitive Power of the WTEG

**Figure 5:**
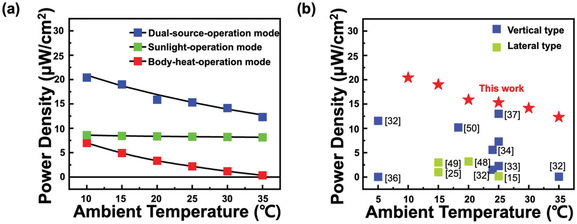
a) Power density of WTEGs in three different modes at various ambient temperatures. b) Comparison of the performance of the TEG prepared in this work with several reported TEGs.

The output power of the WTEG was found to be insensitive to changes in the ambient temperature. Since human skin demonstrates homeostasis, maintaining a steady state while adjusting to conditions, the body temperature remains at ≈36 °C even when the ambient temperature changes. As a result, when the ambient temperature approaches the body temperature, it is theoretically challenging to create a Δ*T*. **Figure** **5**a shows the power density of the WTEG in three different modes as a function of the ambient temperature. In body‐heat‐operation mode, the power density of the WTEG is almost disappeared at the ambient temperature of 35 °C. Our WTEG can overcome this issue to some extent after absorbing additional heat from solar energy. In dual‐source‐operation mode, as the ambient temperature increases from 10 to 35 °C, the power density of the WTEG decreases slightly from 20.41 to 12.32 µW cm^−2^. This is because, as observed from the power density of the WTEG in sunlight‐operation mode, a constant amount of power can be obtained by absorbing sunlight regardless of the ambient temperature. The WTEG in sunlight‐operation mode maintains the power density of 8–8.5 µW cm^−2^ at all ambient temperatures. Compared with other reported lateral or vertical types of flexible WTEGs under actual operating conditions, our WTEG obtains high power at all ambient temperatures due to the additional heat stemming from sunlight absorption (Figure 5b).^[^ ^15^, ^25^, ^32^, ^33^, ^34^, ^36^, ^37^, ^48^, ^49^, ^50^ ^]^ The decrease in power as the ambient temperature approaches 35 °C is mainly caused by the low Δ*T* between the body and ambient temperatures. The Δ*T* from body heat almost disappears, but the Δ*T* from sunlight absorption remains constant when the ambient temperature is 35 °C. Therefore, in the body‐heat‐operation mode, the trend of decreasing power is identical to the trend of the power in the dual‐source‐operation mode, and the power converges to zero when the ambient temperature approaches 35 °C.

### 2.5 Mechanical Properties of the WTEG

**Figure 6:**
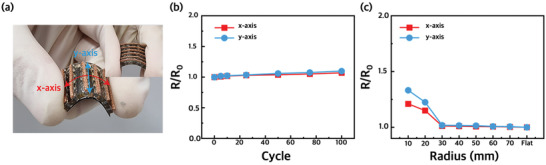
a) Photographs of the TEGs showing excellent flexibility. b) Bending test of the TEGs showing stable electrical resistance during the bending test along two different directions (radius = 50 mm). c) Electrical resistance stability under bending stress with various bending radius. The resistance of the WTEG was measured by reducing the bending radius without release.

For wearable devices, obtaining good mechanical properties is as essential as the TE performance of devices. Since most of the human body is curved, a WTEG attached to the body will inevitably be bent. Regarding flexible WTEGs, the introduction of PDMS prevents each leg from being ruptured by extreme deformation. PDMS firmly adheres to the TE legs, preventing the TE legs from deviating from their positions when bent and dispersing the stress applied to the electrodes. In addition, we introduced an Ag electrode deposited with e‐beam evaporation that perfectly adheres to the PDMS surface. Cu sheets, often used as electrodes in TEGs, show poor adhesion to PDMS, thereby resulting in delamination when bent. Compared to the Cu sheet electrode, the deposited Ag electrode maintains constant electrical resistance after being bent 100 times (Figure S8, Supporting Information). To demonstrate the mechanical robustness of the WTEG, repeated bending tests and stress tests under various bending curvatures were conducted. As shown in **Figure** **6**b, the generator was repeatedly bent up to 100 cycles with a bending radius of 50 mm. Despite the repeated bending cycles, the device exhibits stable internal electrical resistance regardless of the bending direction. When bent 100 times in the *x*‐axis and *y*‐axis directions, the electrical resistance increases by 7% and 9.7%, respectively. Furthermore, there is no significant change in the internal electrical resistance when bending in any direction with an allowable bending radius down to 30 mm (<2%) (Figure 6c). The endurance of the WTEG is slightly poor when bending in the *y*‐axis direction because the Cu foam detaches from the Ag electrode and inflicts damage on the surface during repeated bending due to insufficient adhesion between the Cu foam and Ag electrode. The robustness of the device means that the WTEG remains effective when attached to curved human skin.

## 3 Conclusions

In this study, we proposed a novel structure of sunlight‐assisted vertical‐type WTEG with a high Δ*T* and power density by introducing a solar absorbing layer that brings benefits in various conditions. Without sunlight (body‐heat‐operation mode), WTEG is driven not only by body heat but also by indirect sunlight or artificial light absorbed through a solar absorber, generating ≈35% more power than WTEG without a solar absorber. When the WTEG is exposed to sunlight (dual‐source‐operation mode), the solar absorber attached to the bottom of the WTEG harvests >95% of the available sunlight, increasing the temperature of the hot side. In addition, due to the low thermal conductivity and hot temperature (≈36 °C) of the skin, heat loss from the hot side is reduced, and additional heat is supplied. As a result, the WTEG obtained the highest power density of 15.33 µW cm^−2^ at room temperature. Compared to the power density of only 2.19 µW cm^−2^ in body‐heat‐operation mode, the power density of the dual‐source‐operation mode was seven times higher and is the highest power density among all types of flexible WTEGs reported under actual operating conditions. In addition, the power density of the WTEG was less sensitive to ambient temperature, maintaining a high power density. Generally, when the air temperature approaches body temperature (≈36 °C), the Δ*T* between the body temperature and ambient temperature disappears, resulting in a low power density. However, at an ambient temperature of 35 °C, our WTEG can be operated because the power from sunlight remains constant, even though the power from body heat disappears. Our WTEG can be expected to expand its applications by proposing a new design for high‐performance, adaptable, and durable energy harvesting devices.

## 4 Experimental Section

### Materials

The BiTe based TE legs were purchased from Tesbi (Korea). The composition and thermoelectric properties of the legs are displayed in Table 1. Solar absorber (Pyromark 1200, Tempil), PDMS (Dow Corning), the 99.99%‐pure copper foam (TMAX Inc.), and 9–10 µΩ cm‐resistivity silver paste (Sigma–Aldrich) were used.

### Fabrication Process of the WTEG

The fabrication process of the WTEG is schematically illustrated in Figure 1. First, the p‐n TE legs with a height of 1.5 mm and various cross‐sectional areas were aligned with systemically calculated spacing between adjacent TE legs. Before filling the PDMS, PI films were attached to the bottom and top sides of the aligned TE legs as protecting layers. The PDMS was filled between the TE legs and cured at 110 °C for 1 h. After peeling off the PI films, a 50 nm/4000 nm thick Ni/Ag electrode was deposited on the top of the PDMS using electron beam evaporation to form a top electrode. Before fabricating the bottom electrode, the solar absorber was painted on the 50 µm‐thick PI film (2cm × 2cm) using a brush. Ni/Ag electrodes with the same thickness as the bottom electrode were deposited using an e‐beam evaporator on the PI/Solar absorber film. the bottom electrode/solar absorber/PI film was attached to the bottom of the PDMS while aligning the electrode and TE leg and electrically connecting them together with highly conductive silver paste Finally, the copper foam was attached along the aligned TE legs using thermally conductive epoxy.

### Characterization of the WTEG

The Seebeck coefficients and electrical conductivity of the p‐ and n‐type TE legs were measured using a seebeck coefficient analysis (SBA458, NETSCH). The thermal conductivity (*κ* = ɑC_P_ *ρ*) was calculated from the measurements of the thermal diffusivity (ɑ), specific heat (C_P_), and density(*ρ*) of the bulk TE legs. The density was measured by density test of plastics by displacement (ASTM D792). The specific heat was measured by a differential scanning calorimetry (Q200, TA). The thermal diffusivity was measured by a thermal diffusivity measuring system (LFA 447 NanoFlash, NETZSCH). The thermoelectric characteristics of the TE leg are summarized in Table S1 (Supporting Information). The performance of the WTEG was measured using a home‐made TE measuring system. The WTEG was placed on a copper block with a constant temperature and a polymer with thermal conductivity similar to the body skin to create conditions similar to the actual wearing environment. The Δ*T* between the hot side and cold side was monitored via a thermocouple and IR camera (FLIR Co. Ltd.). The WETG was also measured under 1‐sun sunlight (AM1.5 spectrum, 100 mW cm^−2^) exposure conditions using solar simulation (Class AAA, Oriel Sol3A, Newport). The incident flux was confirmed by a National Renewable Energy Laboratory (NREL)‐verified solar cell (PV Measurements). The electrode of the WTEG was connected to a source meter (Keithley 2400) and the *I*–*V* characteristics were measured using the LabVIEW program after the WTEG reached a thermal equilibrium state. The bending and durability test was performed using the folding tester (JIFT–500). The resistance change of the WTEG during the bending test was measured with a bending radius of 50 mm at a rate of 150 mm min^−1^.

### Simulation of the WTEG

The temperature distribution and electrical properties of the WTEG were predicted using heat transfer and thermoelectric effect simulation models in COMSOL Multiphysics. In order to increase the accuracy of the simulation, the ZT value and efficiency of the TE leg at 300–400 K were measured (Figure S9, Supporting Information). ZT, a dimensionless figure‐of‐merit showing the performance of the TE leg, is defined by Equation (3):

(3) $$ZT\;=\frac{\sigma S^{2}}{\kappa }\;T$$

where, *σ*, *S*, *κ* are electrical conductivity, Seebeck coefficient, and thermal conductivity, respectively. These are summarized in Table S1 (Supporting Information). Theoretical converion efficiency of the TE leg can be obtained from ZT by the Equation (4):

(4) $$\eta_{max}=\frac{\Delta T}{T_{h}}\;\frac{\sqrt{1+ZT_{avg}}-1}{\sqrt{1+ZT_{avg}}+T_{c}/T_{h}}$$

where *T*_c_ is the temperature of the cold side, *T*_h_ is the temperature of the hot side, and *ZT*_avg_ is the average *ZT* value of the TE leg. The electrical resistance of the WTEG was also estimated after connecting the TE legs in series (Figure S6, Supporting Information). The properties of the copper foam and solar absorber were extracted from datasheets provided by TMAX and Tempil, respectively, and the properties of the other materials were taken from the values provided by COMSOL. The output power was derived by $P_{output}=\frac{{V_{OC}}^{2}}{R_{i}{\left(1+R_{L}/R_{i}\right)}^{2}}$, modified from Equation (1). Theoretically, the maximum power is obtained when *R_L_* = *R_i_*, but considering the Peltier effect, we calculated to have the maximum power when $R_{L}=R_{i}\;\sqrt{1+ZT}$.^[^ ^21^ ^]^

## Conflict of Interest

The authors declare no conflict of interest.

## Supporting information

Supporting Information

## Data Availability

The data that support the findings of this study are available from the corresponding author upon reasonable request.

## References

[1] M. Dargusch , W. D. Liu , Z. G. Chen , Adv. Sci. 2020, 7, 2001362. 10.1002/advs.202001362 PMC7509711 32999843

[2] Y. S. Jung , D. H. Jeong , S. B. Kang , F. Kim , M. H. Jeong , K.‐S. Lee , J. S. Son , J. M. Baik , J.‐S. Kim , K. J. Choi , Nano Energy 2017, 40, 663.

[3] H. Choi , S. J. Kim , Y. Kim , J. H. We , M.‐W. Oh , B. J. Cho , J. Mater. Chem. C 2017, 5, 8559.

[4] Z. Cao , E. Koukharenko , M. J. Tudor , R. N. Torah , S. P. Beeby , Sens. Actuators, A 2016, 238, 196.

[5] C. Dun , W. Kuang , N. Kempf , M. Saeidi‐Javash , D. J. Singh , Y. Zhang , Adv. Sci. 2019, 6, 1901788. 10.1002/advs.201901788 PMC6891908 31832319

[6] N. Kim , S. Lienemann , I. Petsagkourakis , D. Alemu Mengistie , S. Kee , T. Ederth , V. Gueskine , P. Leclère , R. Lazzaroni , X. Crispin , K. Tybrandt , Nat. Commun. 2020, 11, 1424. 32188853 10.1038/s41467-020-15135-w PMC7080746

[7] J. F. Serrano‐Claumarchirant , I. Brotons‐Alcázar , M. Culebras , M. J. Sanchis , A. Cantarero , R. Muñoz‐Espí , C. M. Gómez , ACS Appl. Mater. Interfaces 2020, 12, 46348. 32965099 10.1021/acsami.0c12076

[8] D. Qu , X. Li , H. Wang , G. Chen , Adv. Sci. 2019, 6, 1900584. 10.1002/advs.201900584 PMC6685505 31406671

[9] S. D. Xu , M. Hong , M. Li , Q. Sun , Y. Yin , W. D. Liu , X. L. Shi , M. Dargusch , J. Zou , Z. G. Chen , Appl. Phys. Rev. 2021, 8, 041404.

[10] Y. Wang , M. Hong , W.‐D. Liu , X.‐L. Shi , S.‐D. Xu , Q. Sun , H. Gao , S. Lu , J. Zou , Z.‐G. Chen , Chem. Eng. J. 2020, 397, 125360.

[11] K. Nan , S. D. Kang , K. Li , K. J. Yu , F. Zhu , J. Wang , A. C. Dunn , C. Zhou , Z. Xie , M. T. Agne , H. Wang , H. Luan , Y. Zhang , Y. Huang , G. J. Snyder , J. A. Rogers , Sci. Adv. 2018, 4, eaau5849. 30406207 10.1126/sciadv.aau5849 PMC6214638

[12] V. Karthikeyan , J. U. Surjadi , J. C. K. Wong , V. Kannan , K.‐H. Lam , X. Chen , Y. Lu , V. A. L. Roy , J. Power Sources 2020, 455, 227983.

[13] M. H. Jeong , A. Sanger , S. B. Kang , Y. S. Jung , I. S. Oh , J. W. Yoo , G. H. Kim , K. J. Choi , J. Mater. Chem. A 2018, 6, 15621.

[14] G. H. Kim , L. Shao , K. Zhang , K. P. Pipe , Nat. Mater. 2013, 12, 719. 23644522 10.1038/nmat3635

[15] Q. Zhou , K. Zhu , J. Li , Q. Li , B. Deng , P. Zhang , Q. Wang , C. Guo , W. Wang , W. Liu , Adv. Sci. 2021, 8, 2004947. 10.1002/advs.202004947 PMC8224459 34194935

[16] S. D. Xu , X. L. Shi , M. Dargusch , C. A. Di , J. Zou , Z. G. Chen , Prog. Mater. Sci. 2021, 121, 100840.

[17] J.‐Y. Kim , W. Lee , Y. H. Kang , S. Y. Cho , K.‐S. Jang , Carbon 2018, 133, 293.

[18] Y. Ryu , L. Yin , C. Yu , J. Mater. Chem. 2012, 22, 6959.

[19] G. Wu , Z.‐G. Zhang , Y. Li , C. Gao , X. Wang , G. Chen , ACS Nano 2017, 11, 5746. 28511002 10.1021/acsnano.7b01279

[20] Q. Hu , Z. Lu , Y. Wang , J. Wang , H. Wang , Z. Wu , G. Lu , H.‐L. Zhang , C. Yu , J. Mater. Chem. A 2020, 8, 13095.

[21] Y. Wang , Z. Lu , Q. Hu , X. Qi , Q. Li , Z. Wu , H.‐L. Zhang , C. Yu , H. Wang , J. Mater. Chem. A 2021, 9, 3341.

[22] J. Liu , B. Van Der Zee , R. Alessandri , S. Sami , J. Dong , M. I. Nugraha , A. J. Barker , S. Rousseva , L. Qiu , X. Qiu , N. Klasen , R. C. Chiechi , D. Baran , M. Caironi , T. D. Anthopoulos , G. Portale , R. W. A. Havenith , S. J. Marrink , J. C. Hummelen , L. J. A. Koster , Nat. Commun. 2020, 11, 5694. 33173050 10.1038/s41467-020-19537-8 PMC7655812

[23] J. Liu , L. Qiu , G. Portale , M. Koopmans , G. Ten Brink , J. C. Hummelen , L. J. A. Koster , Adv. Mater. 2017, 29, 1701641. 10.1002/adma.201701641 28722288

[24] J. Liu , L. Qiu , G. Portale , S. Torabi , M. C. A. Stuart , X. Qiu , M. Koopmans , R. C. Chiechi , J. C. Hummelen , L. J. Anton Koster , Nano Energy 2018, 52, 183.

[25] S. J. Kim , J. H. We , B. J. Cho , Energ. Environ. Sci. 2014, 7, 1959.

[26] B. Zhu , X. Liu , Q. Wang , Y. Qiu , Z. Shu , Z. Guo , Y. Tong , J. Cui , M. Gu , J. He , Energ. Environ. Sci. 2020, 13, 2106.

[27] G. Yang , R. Niu , L. Sang , X. Liao , D. R. G. Mitchell , N. Ye , J. Pei , J. F. Li , X. Wang , Adv. Energy Mater. 2020, 10, 2000757.

[28] H. L. Zhuang , J. Pei , B. Cai , J. Dong , H. Hu , F. H. Sun , Y. Pan , G. J. Snyder , J. F. Li , Adv. Funct. Mater. 2021, 31, 2009681.

[29] S. I. Kim , K. H. Lee , H. A. Mun , H. S. Kim , S. W. Hwang , J. W. Roh , D. J. Yang , W. H. Shin , X. S. Li , Y. H. Lee , G. J. Snyder , S. W. Kim , Science 2015, 348, 109. 25838382 10.1126/science.aaa4166

[30] W. D. Liu , D. Z. Wang , Q. F. Liu , W. Zhou , Z. P. Shao , Z. G. Chen , Adv. Energy Mater. 2020, 10, 2000367.

[31] B. Lee , H. Cho , K. T. Park , J.‐S. Kim , M. Park , H. Kim , Y. Hong , S. Chung , Nat. Commun. 2020, 11, 5948. 33230141 10.1038/s41467-020-19756-z PMC7684283

[32] F. Suarez , D. P. Parekh , C. Ladd , D. Vashaee , M. D. Dickey , M. C. Ozturk , Appl. Energ. 2017, 202, 736.

[33] C. S. Kim , G. S. Lee , H. Choi , Y. J. Kim , H. M. Yang , S. H. Lim , S.‐G. Lee , B. J. Cho , Appl. Energ. 2018, 214, 131.

[34] H. Park , D. Lee , D. Kim , H. Cho , Y. Eom , J. Hwang , H. Kim , J. Kim , S. Han , W. Kim , J. Phys. D: Appl. Phys. 2018, 51, 365501.

[35] S. J. Kim , H. E. Lee , H. Choi , Y. Kim , J. H. We , J. S. Shin , K. J. Lee , B. J. Cho , ACS Nano 2016, 10, 10851. 28024371 10.1021/acsnano.6b05004

[36] P. Yang , K. Liu , Q. Chen , X. Mo , Y. Zhou , S. Li , G. Feng , J. Zhou , Angew. Chem., Int. Ed. 2016, 55, 12050. 10.1002/anie.201606314 27557890

[37] C. S. Kim , H. M. Yang , J. Lee , G. S. Lee , H. Choi , Y. J. Kim , S. H. Lim , S. H. Cho , B. J. Cho , ACS Energy Lett. 2018, 3, 501.

[38] H. Liu , Y. Wang , D. Mei , Y. Shi , Z. Chen , Springer, Singapore, 2017

[39] S. Hong , Y. Gu , J. K. Seo , J. Wang , P. Liu , Y. S. Meng , S. Xu , R. K. Chen , Sci. Adv. 2019, 5, eaaw0536. 31114803 10.1126/sciadv.aaw0536 PMC6524982

[40] Y. Shi , Y. Wang , D. Mei , Z. Chen , Ieee Access 2018, 6, 43602.

[41] Y. G. Lee , J. Kim , M.‐S. Kang , S.‐H. Baek , S. K. Kim , S.‐M. Lee , J. Lee , D.‐B. Hyun , B.‐K. Ju , S. E. Moon , J.‐S. Kim , B. Kwon , Adv. Mater. Technol. 2017, 2, 1600292.

[42] S. Fan , Y. Gao , A. Rezania , Renewable Energy 2021, 173, 581.

[43] F. Suarez , A. Nozariasbmarz , D. Vashaee , M. C. Öztürk , Energ. Environ. Sci. 2016, 9, 2099.

[44] R. Riemer , A. Shapiro , J. NeuroEng. Rehabil. 2011, 8, 22. 21521509 10.1186/1743-0003-8-22 PMC3098156

[45] C. K. Ho , A. R. Mahoney , A. Ambrosini , M. Bencomo , A. Hall , T. N. Lambert , J. Sol. Energy Eng. 2014, 136, 014502.

[46] M. Lossec , B. Multon , H. Ben Ahmed , C. Goupil , EPJ Appl. Phys. 2010, 52, 11103.

[47] J. Yuan , R. Zhu , Appl. Energ. 2020, 271, 115250.

[48] Y. Eom , D. Wijethunge , H. Park , S. H. Park , W. Kim , Appl. Energ. 2017, 206, 649.

[49] T. Nguyen Huu , T. Nguyen Van , O. Takahito , Appl. Energ. 2018, 210, 467.

[50] M. Hyland , H. Hunter , J. Liu , E. Veety , D. Vashaee , Appl. Energ. 2016, 182, 518.

