# Supplementary material for: Operation of Wearable Thermoelectric Generators Using Dual Sources of Heat and Light
Source: Adv Sci (Weinh). 2022 Feb 24;9(12):2104915. doi: 10.1002/advs.202104915 (PMC9036048; doi:10.1002/advs.202104915)
Supplement: Supplementary file 1 — Supporting Information [file ADVS-9-2104915-s001.pdf]

ADVANCED SCIENCE
Open Access

Supporting Information

for *Adv. Sci.*, DOI 10.1002/advs.202104915

Operation of Wearable Thermoelectric Generators Using Dual Sources of Heat and Light

*Myeong Hoon Jeong, Kwang-Chon Kim, Jin-Sang Kim and Kyoung Jin Choi**

Operation of wearable thermoelectric generators using dual sources of heat and light

Myeong Hoon Jeong[a], *Kwang-Chon Kim*[b], *Jin-Sang Kim*[c], *Kyoung Jin Choi*[a] *

[a] Department of Materials Science and Engineering, Ulsan National Institute of Science and Technology, Ulsan 44919, Republic of Korea

[b] Center for Electronic Materials, Korea Institute of Science and Technology, Seoul 02792, Republic of Korea

[c] KIST Jeonbuk Institute of Advanced Composite Materials, Wanju-gun 55324, Republic of Korea

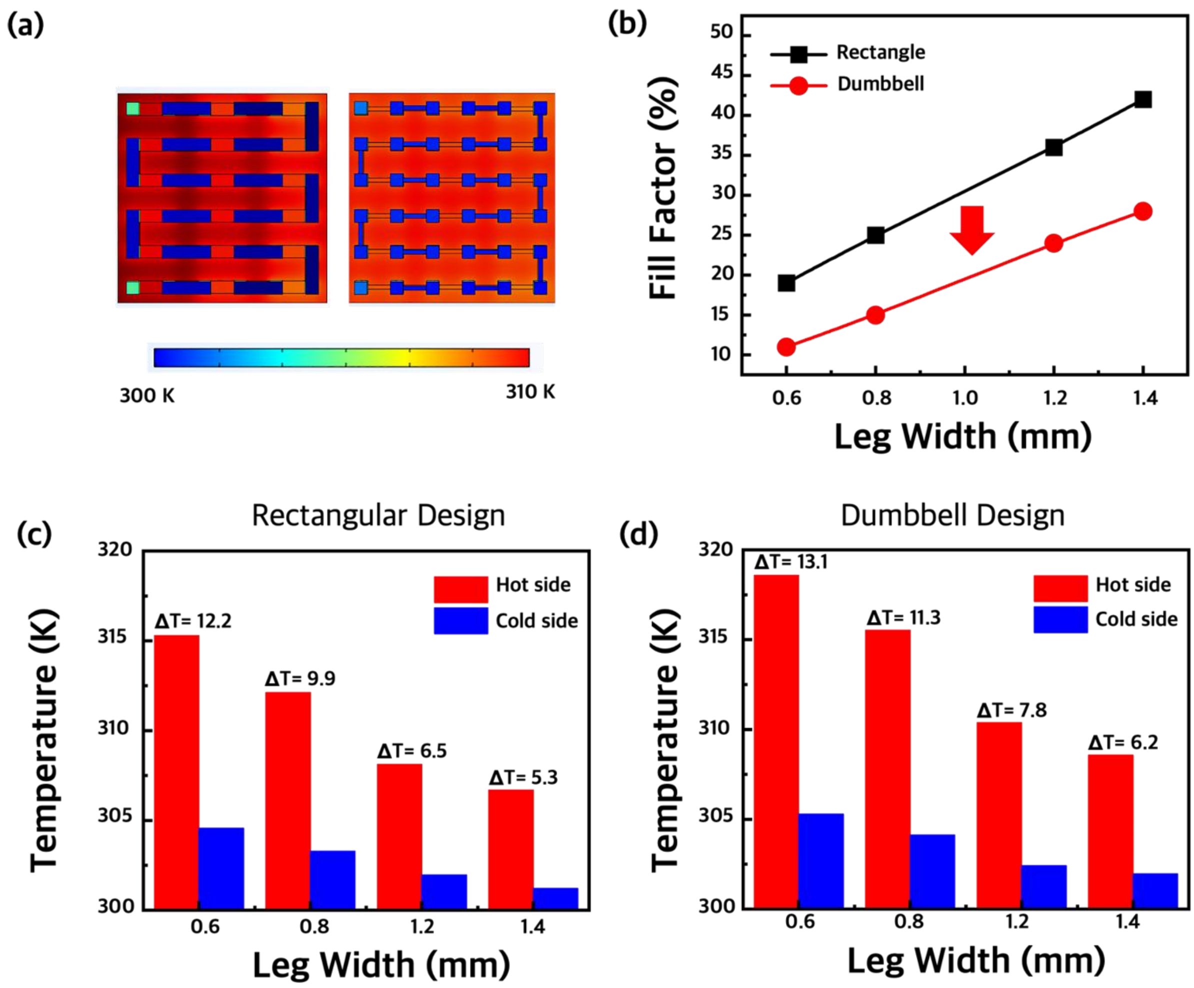

Fig. S1 (a) Comparison of the temperature distributions of TEGs with different electrode shapes. (b) The fill factor of the TE leg and electrode changes with the different electrode shapes. Simulated temperatures on the cold and hot sides with the application of (c) rectangular and (d) dumbbell electrode designs.

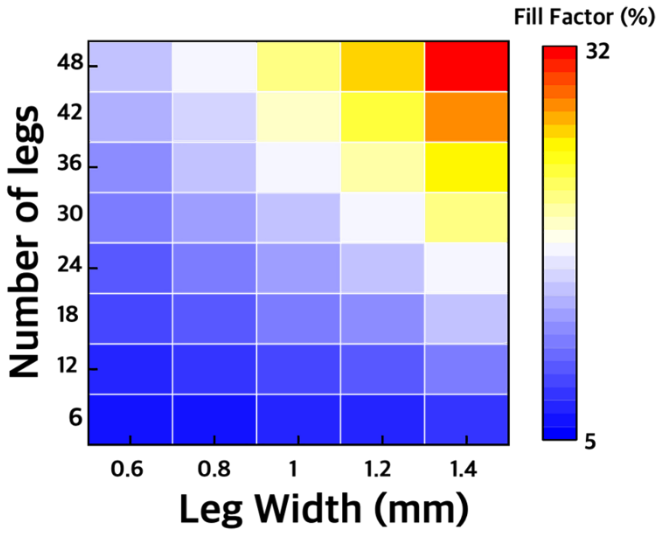

Fig. S2 Calculated fill factor of the TE leg and electrode depending on the various leg widths and number of TE legs.

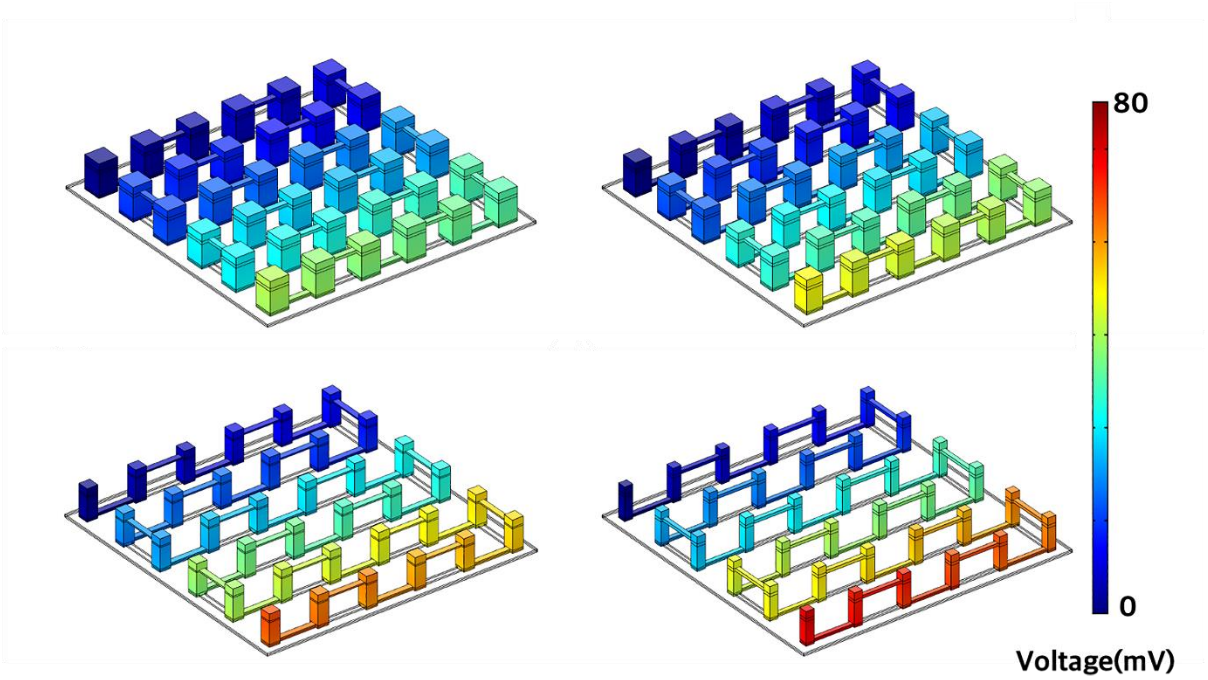

Fig. S3 (a) Simulation results showing the open-circuit voltage of the TEGs with TE legs of different dimensions.

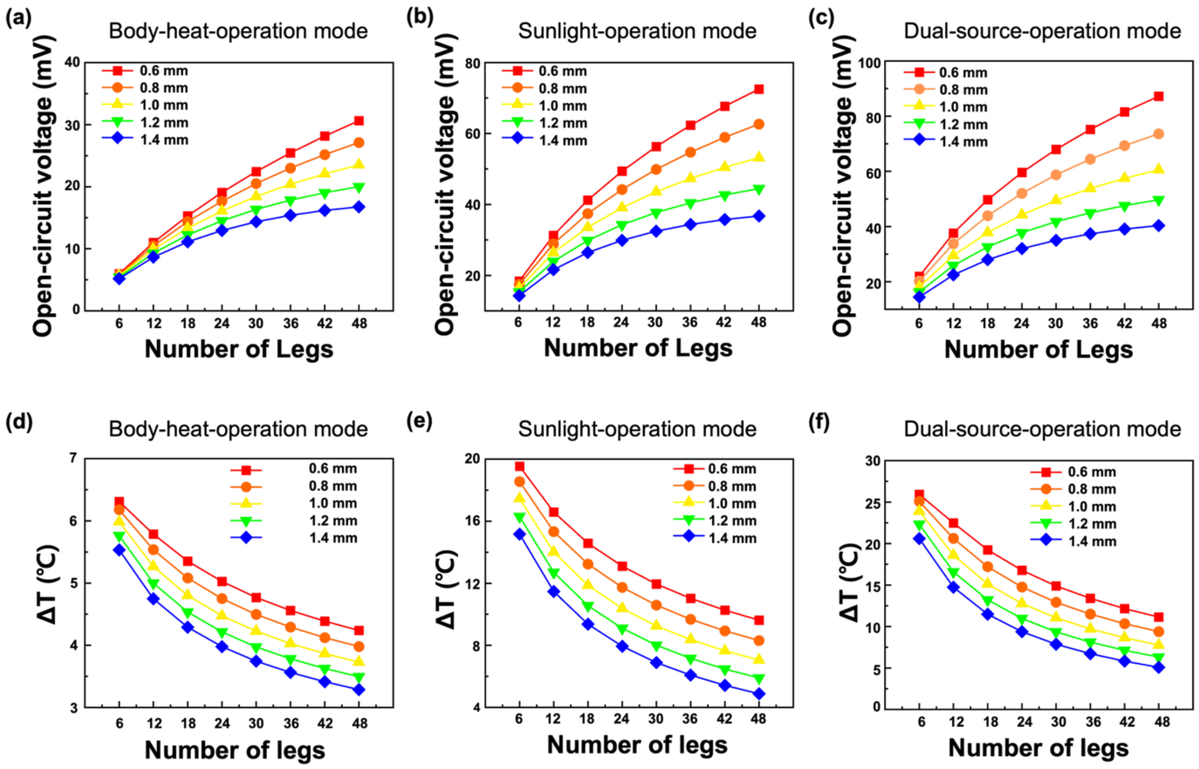

Fig. S4. V_{OC} and ΔT of the WTEG (a)-(d) in body-heat-operation mode, (b)-(e) sunlight-operation mode, and (c)-(f) dual-source-operation mode depending on the number of legs.

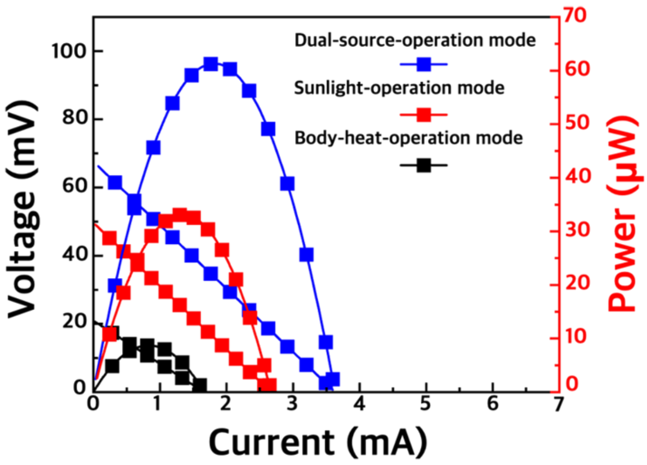

Fig. S5 Measured TEG performance including voltage and power as a function of current.

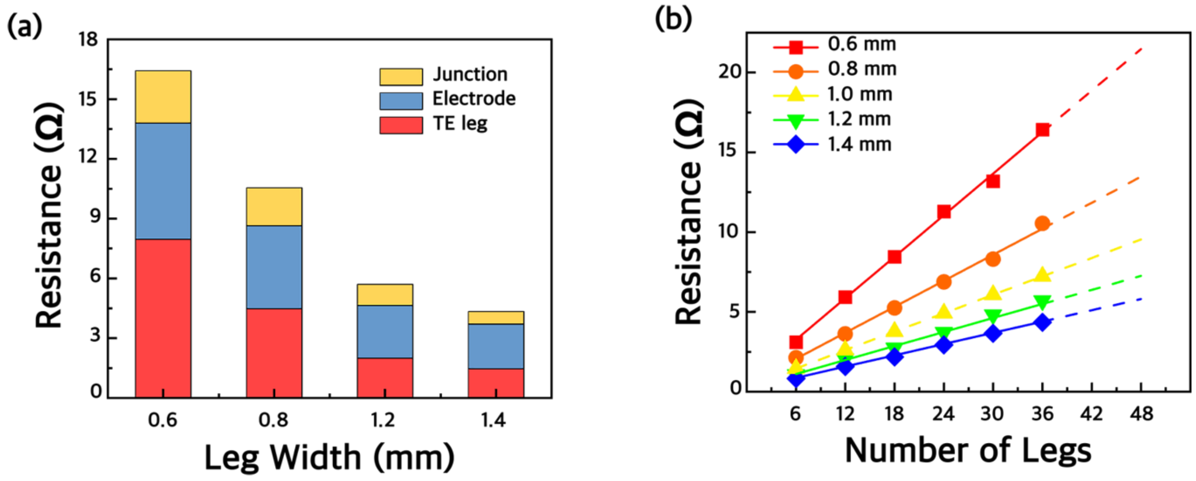

Fig. S6 (a) Components of the total electrical resistance in WTEGs with 36 legs. (b) Measured electrical resistance change of the WTEGs as a function of the number of legs. The total electrical resistance was predicted by measuring the resistance change as the number of legs increased.

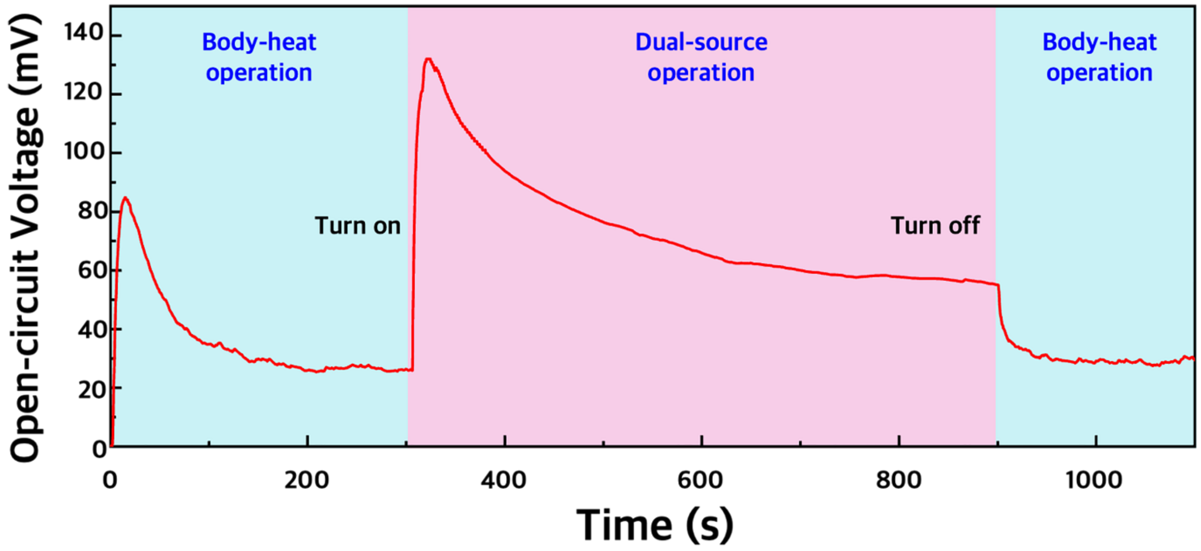

Fig. S7 Measurement of V_{OC} of WTEG over time. When the WTEG is attached to the body, the V_{OC} increases immediately. Under dual-source operation mode, V_{OC} increases dramatically due to thermal energy from sunlight and reaches equilibrium. After turning off sunlight, the V_{OC} decreases

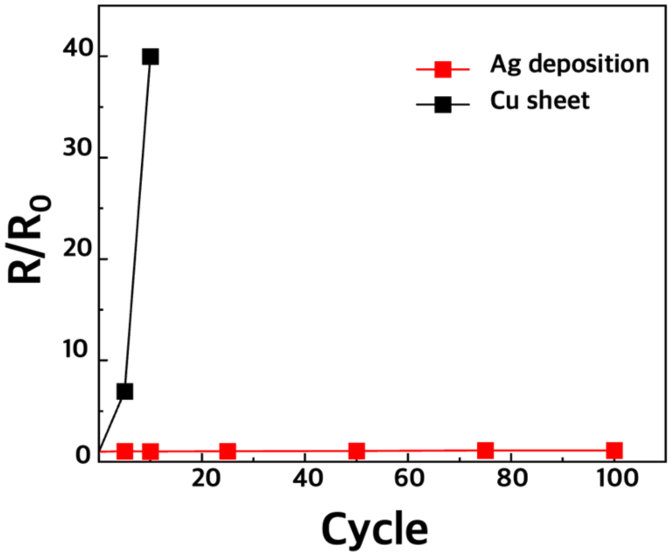

Fig. S8 Bending test of the TEGs showing the change in electrical resistance during the bending test with different electrode types.

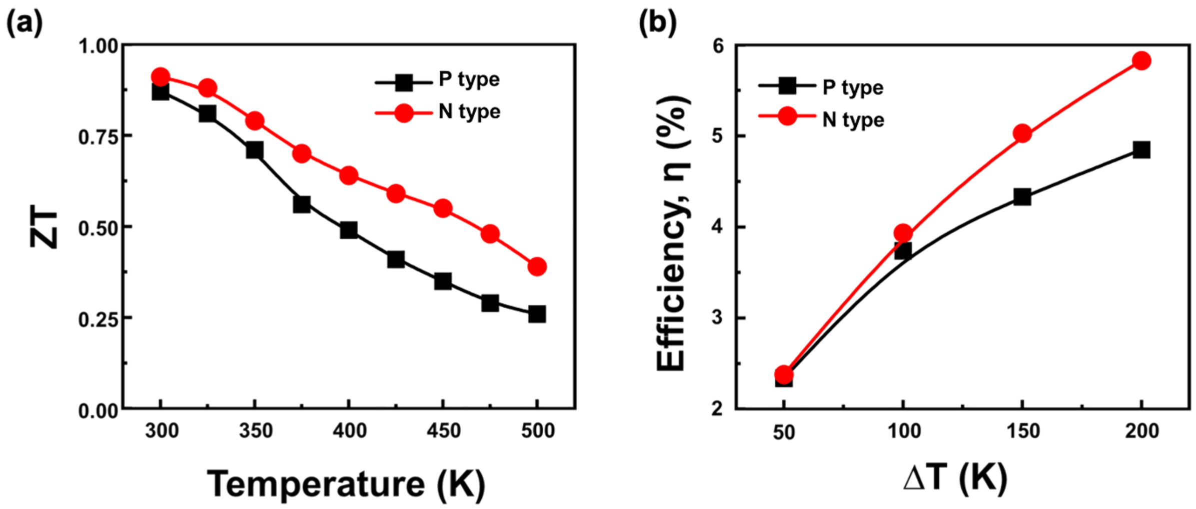

Fig. S9 (a) ZT of the p-type and n-type TE legs as a function of the temperature. (b) Theoretical conversion efficiency of the p-type and n-type TE legs obtained from the ZT.

	**Seebeck coefficient (μV/K)**	**Electrical conductivity (S/m)**	**Thermal conductivity (W/m K)**	**ZT value (@300K)**
***P* type Bi_2Te_3**	210±10	79000±6000	1.25	0.87
***N* type Bi_2Te_3**	218±8	76500±5500	1.21	0.91

Table S1. Thermoelectric properties of TE legs (All parameters were obtained at 300 K.)
